# Computational identification of genes modulating stem height–diameter allometry

**DOI:** 10.1111/pbi.12579

**Published:** 2016-06-15

**Authors:** Libo Jiang, Meixia Ye, Sheng Zhu, Yi Zhai, Meng Xu, Minren Huang, Rongling Wu

**Affiliations:** ^1^ Center for Computational Biology College of Biological Sciences and Technology Beijing Forestry University Beijing China; ^2^ Co‐Innovation Center for Sustainable Forestry in Southern China Nanjing Forestry University Nanjing China; ^3^ Center for Statistical Genetics The Pennsylvania State University Hershey PA USA

**Keywords:** height–diameter allometry, mathematical equation, functional mapping, quantitative trait loci

## Abstract

The developmental variation in stem height with respect to stem diameter is related to a broad range of ecological and evolutionary phenomena in trees, but the underlying genetic basis of this variation remains elusive. We implement a dynamic statistical model, functional mapping, to formulate a general procedure for the computational identification of quantitative trait loci (QTLs) that control stem height–diameter allometry during development. Functional mapping integrates the biological principles underlying trait formation and development into the association analysis of DNA genotype and endpoint phenotype, thus providing an incentive for understanding the mechanistic interplay between genes and development. Built on the basic tenet of functional mapping, we explore two core ecological scenarios of how stem height and stem diameter covary in response to environmental stimuli: (i) trees pioneer sunlit space by allocating more growth to stem height than diameter and (ii) trees maintain their competitive advantage through an inverse pattern. The model is equipped to characterize ‘pioneering’ QTLs (*pi* QTLs) and ‘maintaining’ QTLs (*mi* QTLs) which modulate these two ecological scenarios, respectively. In a practical application to a mapping population of full‐sib hybrids derived from two *Populus* species, the model has well proven its versatility by identifying several *pi* QTLs that promote height growth at a cost of diameter growth and several *mi* QTLs that benefit radial growth at a cost of height growth. Judicious application of functional mapping may lead to improved strategies for studying the genetic control of the formation mechanisms underlying trade‐offs among quantities of assimilates allocated to different growth parts.

## Introduction

The allocation pattern of stem growth to height and diameter is a structural characteristic of a tree that reflects its capacity to adapt to various environmental conditions (Feldpausch *et al*., 2011; Franco and Kelly, 1998; Niklas and Spatz, 2004; Price *et al*., 2007). As a key element of stem form, the allometric relationship of stem height and diameter determines the volume of the harvestable stem and carbon storage and is also thought to be associated with wood structure, for example lignin and cellulose content (Voelker *et al*., 2011a,b; Waghorn *et al*., 2007). Differences in the height–diameter relationship arise from differences in biomass allocation throughout the stem, affected by growth conditions, such as spacing, biomechanical factors, site quality and age (Feldpausch *et al*., 2011; Ilomaki *et al*., 2003; Niklas, 1995). It has been observed that the relationship between tree height and diameter varies substantially along spatial and environmental gradients and that such pronounced differences also occur at the species, population, family and genotype levels. For example, light‐demanding (pioneer) species typically have a slender stem, assisting them to quickly attain or maintain position in the canopy (Harrington and DeBell, 1996; King, 1990; Poorter *et al*., 2003). Populations in higher altitudes that encounter frequently high winds and snows might evolve stouter stems and crowns to withstand those extreme climates than those from the same species in lower altitudes (Feldpausch *et al*., 2011; Hulshof *et al*., 2015; Scarano, 2002).

With these discoveries of variation as environmental adaptation, a question naturally arises about the genetic dissection of stem form (Andersson *et al*., 2007; Niklas and Marler, 2007). Kroon *et al*. (2008) used 10 diallel progeny trials of Scots pine to estimate the narrow‐sense heritability of 0.22 for the ratio of stem height to diameter usually served as a measure of stem form (Niklas, 1995). A few studies reported on the detection of quantitative trait loci (QTLs) that affect stem forms using molecular markers. Several QTLs were found to affect stem height–diameter ratio in poplar (Wu, 1998). Similar stem form QTLs were identified in *Salix* (Tsarouhas *et al*., 2002) and mei, an ornamental woody plant (Sun *et al*., 2014). However, these studies were based on the static measure of stem form, which has two limitations. First, it does not capture a dynamic process of stem height growth that varies with radial growth during ontogeny. Second, there is no mechanistic underpinning revealed in the allometric scaling of height vs. diameter.

For light‐like trees like poplars, rigorous height growth is essential in a competitive environment, because this helps them obtain a better arrangement of leaves up to a sunlit space (Falster and Westoby, 2003; Petit and Hampe, 2006; Poorter *et al*., 2006). In order to maintain their competitive merit, trees invest more energy to stem diameter growth through increasing leaf mass (Kantola and Makela, 2004; Makela and Vanninen, 2001). The biological principle behind the height–diameter relationship can be reflected by deriving mathematical equations (Henry and Aarssen, 1999; Huang *et al*., 1992; Hulshof *et al*., 2015). We argue that the integration of these mathematical equations into functional mapping, a dynamic model of QTL mapping using mathematical aspects of trait formation and development (Li and Wu, 2010; Ma *et al*., 2002; Wu and Lin, 2006; Wu *et al*., 2004; Li & Sillanpaa, 2015), can facilitates our understanding of the developmental genetic architecture of height–diameter relationship in forest trees.

In living systems, biological traits are conferred a function to alter their phenotypes in better response to environmental or developmental signals, during which there must exist a particular set of genes that regulate or reflect such alteration (Gilchrist and Nijhout, 2001; Salazar‐Ciudad and Jernvall, 2010; Wang *et al*., 2005). The estimation of the temporal pattern of genetic regulation allows us to assess the dynamic interplay of genes and development. This question of fundamental importance that remains unanswered from conventional genetic mapping has become tractable through functional mapping. Specific genes that act by changing their effects during development have been identified for age‐specific body mass index (Das *et al*., 2011) and mouse body growth (Wu *et al*., 2004, 2005). Through extensive simulation in previous studies (Li and Sillanpaa, 2013), increased power of detecting significant genes was observed using functional mapping than single‐trait analysis. This finding, from a methodological perspective, strongly argues for the immediate application of functional mapping to dynamic height–diameter allometry. In this article, we implement functional mapping to study the genetic architecture of how height and diameter covary during development and further explore its application to map and identify QTLs for this covariation by analysing a practical mapping data for a forest tree.

## Results

### Mathematical modelling of height–diameter allometry

**Figure 1:**
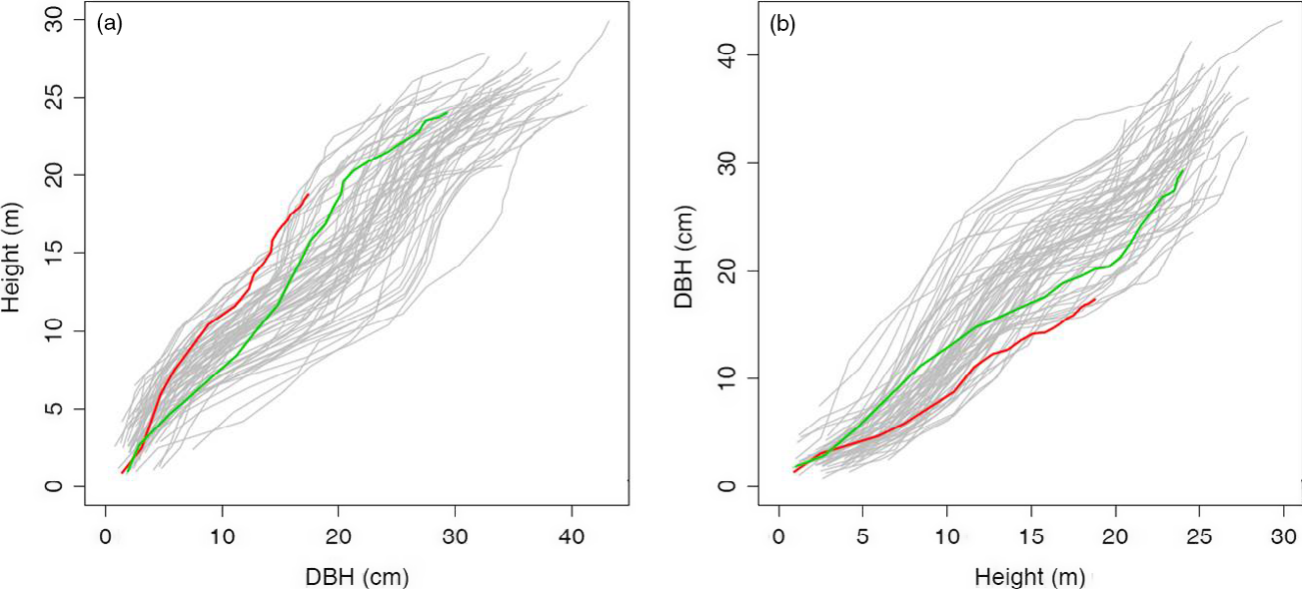
Height–diameter developmental allometry for two parents, I‐69 (green line) and I‐45 (red line), and their progeny (grey line). (a) The allometric changes in stem height as a function of stem diameter. (b) The allometric change in stem diameter as a function of stem height.

Through stem analysis, we obtained the annual growth data of stem height (H) and diameter (D) at stem base in a full‐sib family of *Populus* during the first 24 growing seasons. The developmental pattern of H‐D allometry was plotted in Figure 1, where two parents, I‐69 and I‐45, were observed to display a dramatic difference in the H‐D allometry over development. The trunk of small‐sized I‐45 is consistently more slender in ontogeny than that of large‐sized I‐69, and the progeny derived from these two parents produce remarkable segregation in both stem height and diameter and their developmental relationship. Considerable variation in the H‐D relationship trajectory among progeny reveals possible involvement of specific genes that control this trait. By comparing a series of commonly used H‐D equations given in Huang *et al*. (1992), we finally found that equations (1a) and (1b) are the most parsimonious ones that best fit H‐D relationships in polar hybrids. Thus, by estimating power coefficients (*a*_*H*←*D*_, *b*_*H*←*D*_) and (*a*_*D*←*H*_, *b*_*D*←*H*_) from these equations, we can illustrate how stem height scales with stem diameter and how stem diameter scales with stem height, respectively.

### QTLs for height–diameter allometry

By integrating equations (1a) and (1b) into functional mapping, we test how a QTL controls allometric scaling of height and diameter during ontogeny. The integration of each equation can reveal a different view of ecological significance underlying the genetic control of height–diameter allometry. Equation (1a) describes an ecological process of how stem diameter invests stem height in an efficient way to capture spatial advantage in a competitive environment. Given the same growth of stem diameter, a taller height has a better capacity to increase the exposure of leaves to light, increase the shading of competitors and elevate reproductive or dispersal organs than a shorter height. Significant genes that control the change in height growth with diameter growth detected by equation (1a) are called ‘pioneering QTLs’ (or *pi*QTLs), because they may play a pioneering role in activating trees to capture spatial resources for the optimal fitness of the future.

Equation (1b) specifies a different ecological process in which spatial advantage can be maintained by investing radial growth. Among those trees of the same height, stout ones can preserve the potential of growth towards next stages of competition than slender ones. Those significant genes that determine the increasing amount of radial growth resulting from height growth detected by equation (1b) are named ‘maintaining QTLs’ (or *mi*QTLs) in terms of their role in maintaining the spatial advantage of trees in a competing environment. The identification of *pi*QTLs and *mi*QTLs helps to interpret the mechanistic basis of H‐D allometry in response to environmental perturbations.

#### PiQTLs: How height growth scales with diameter growth

**Figure 2:**
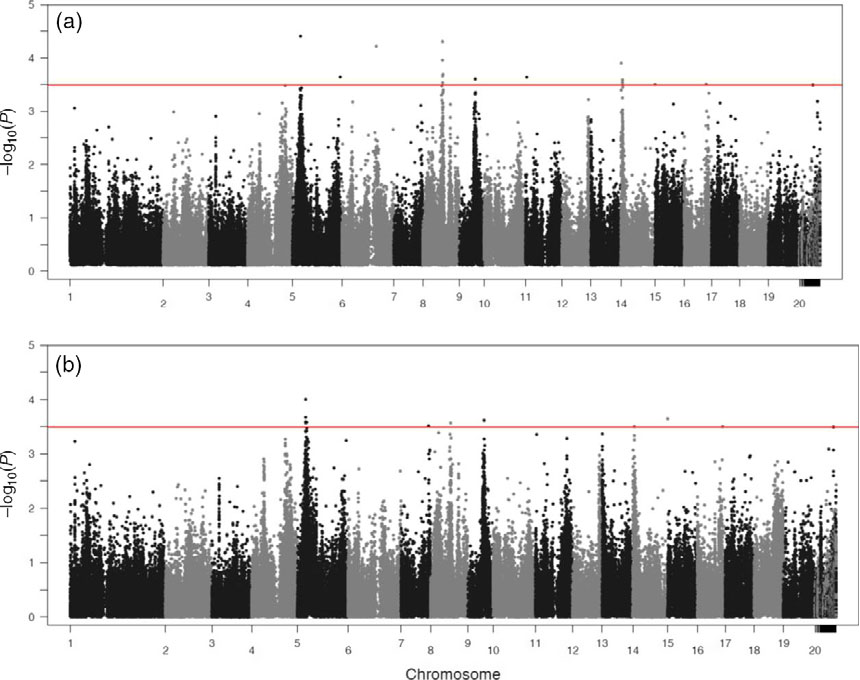
Manhattan plots of −log(*p*) values over the Populus genome. (a) *pi* QTLs for the allometric scaling of height with diameter. (b) *mi* QTLs for the allometric scaling of diameter with height. The red horizontal line is the genomewide critical threshold at the 1% significance level determined through Bonferroni correction.

**Figure 3:**
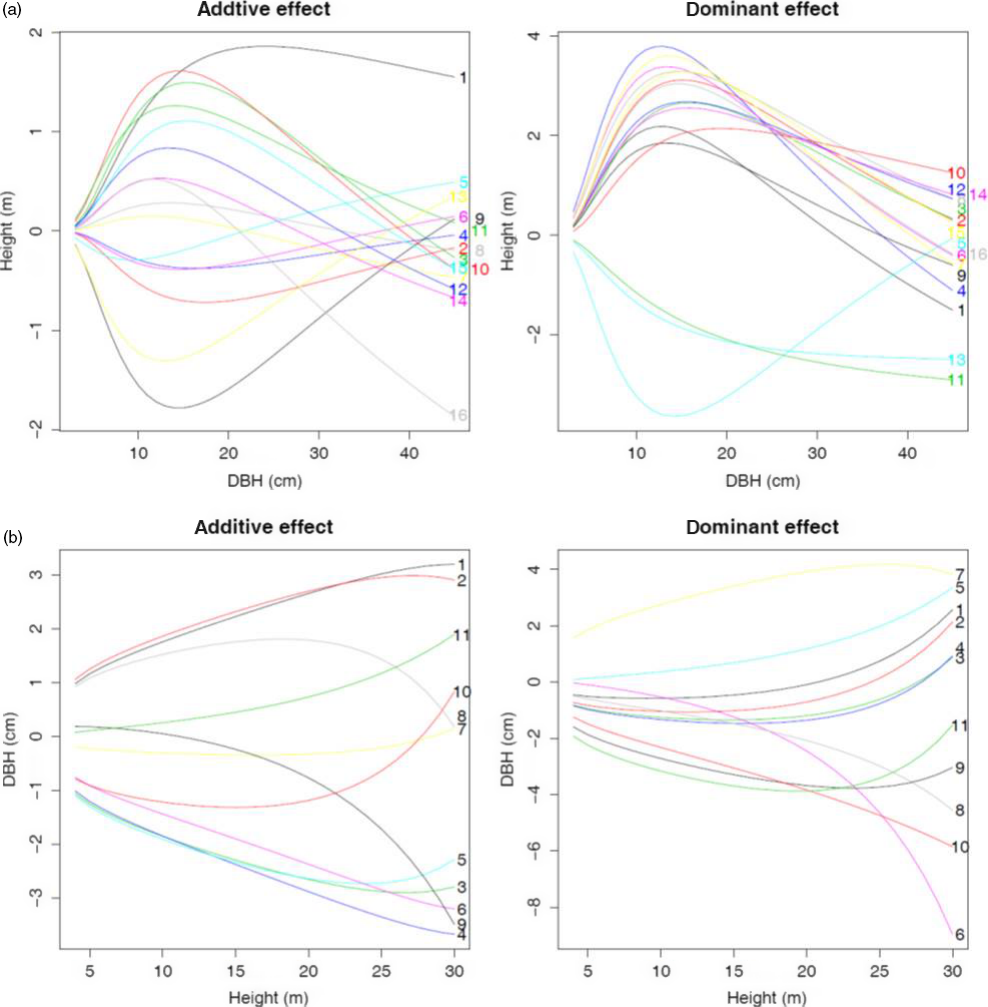
Temporal patterns of additive and dominant genetic effects by significant *pi* QTLs (a) and *mi* QTLs (b).

Functional mapping based on equation (1a) leads to the detection of 16 significant *pi*QTLs responsible for the way how stem height growth is determined by the growth of stem diameter (Table 1; Figure 2a). These *pi*QTLs are distributed on different regions, one on chromosome 6, 9, 11 and 16, two on chromosome 5 and five on chromosome 8 and 14, respectively. As shown in Figure S1, genotypic differences in H‐D curves by equation (1a) have remarkable goodness of fit to the raw data. Different genotypes at each *pi*QTL detected vary in the pattern of how height scales with diameter, and also different *pi*QTLs exemplify different patterns of allometric scaling. Figure 3a illustrates how different *pi*QTLs affect the change in stem height growth as a function of stem diameter. It seems that both additive and dominant effects have been at work, following a similar pattern of determining height–diameter allometry. Although the direction of genetic effects may be different, all *pi*QTLs display an increasing influence on the height–diameter relationship during the early stage of growth. When trees achieve a certain size, say 11 cm in stem diameter, genetic effects decrease with increasing tree size.

**Table 1:** A list of *pi*QTLs for the allometry of tree height with stem diameter and *mi*QTLs for the allometry of stem diameter with tree height in a full‐sib family of interspecific hybrids

$H\left(t\right)=H_{0}+e^{a_{H\leftarrow D}+\frac{1}{b_{H\leftarrow D}+D\left(t\right)}}$							
No.	*pi*QTL	Chr.	Position	Type	Alleles	*P*‐value	Biological function
1	5/4226091	5	4226091	Intercross	C/T	3.90 × 10^−5^	Intergenic
2	5/24780848	5	24780848	Intercross	G/A	2.28 × 10^−4^	Intergenic, POPTR_0005s26940.1 LSD1‐like 1 AT1G62830.1
3	6/17682099	6	17682099	Intercross	G/T	6.00 × 10^−5^	Intergenic
4	8/10141590	8	10141590	Intercross	T/C	1.10 × 10^−4^	Intergenic, POPTR_0008s15160.1 AT5G43150.1
5	8/10163499	8	10163499	Intercross	G/T	4.90 × 10^−5^	Intergenic, POPTR_0008s15190.1 Ribosomal protein S24e family AT5G28060.1
6	8/10214577	8	10214577	Intercross	C/A	2.91 × 10^−4^	Intron, POPTR_0008s15320.1 O‐acetylserine (thiol) lyase (OAS‐TL) isoform A1 AT4G14880.1
7	8/10214969	8	10214969	Intercross	A/G	2.19 × 10^−4^	Intron, POPTR_0008s15320.1 O‐acetylserine (thiol) lyase (OAS‐TL) isoform A1 AT4G14880.1
8	8/10386508	8	10386508	Intercross	T/C	2.03 × 10^−4^	Intergenic
9	9/8371476	9	8371476	Intercross	A/T	2.48 × 10^−4^	Intergenic, POPTR_0009s09470.1 Protein phosphatase 2C family AT4G33920.1
10	11/645420	11	645420	Intercross	G/A	2.30 × 10^−5^	Intergenic
11	14/139257	14	139257	Intercross	T/G	1.25 × 10^−4^	Exon, POPTR_0014s00330.1 Polyketide cyclase/dehydrase and lipid transport superfamily protein AT3G13062.2
12	14/763510	14	763510	Intercross	G/T	2.89 × 10^−4^	Intergenic
13	14/791796	14	791796	Intercross	C/T	2.92 × 10^−4^	Intron, POPTR_0014s00860.1 P‐loop containing nucleoside triphosphate hydrolases superfamily protein AT2G22870.1
14	14/807942	14	807942	Intercross	T/A	2.59 × 10^−4^	Intergenic, POPTR_0014s00880.1 Uncharacterized conserved protein UCP031279 AT1G58420.1
15	14/17660801	14	17660801	Intercross	A/G	0.000313	Intergenic
16	16/11391387	16	11391387	Intercross	C/G	0.000310	Intergenic

$D\left(t\right)=\frac{1-\left[\ln\left(H\left(t\right)-H_{0}\right)-a_{D\leftarrow H}\right]b_{D\leftarrow H}}{\ln\left(H\left(t\right)-H_{0}\right)-a_{D\leftarrow H}}$							
	*mi*QTL	Chr.	Position	Type	Alleles	*P*‐value	Function
1	5/4220981	5	4220981	Intercross	T/C	2.56 × 10^−4^	Intergenic, POPTR_0005s06350.1 Disease resistance protein (TIR‐NBS class), putative AT4G16990.2
2	5/4221532	5	4221532	Intercross	G/C	2.68 × 10^−4^	Intergenic
3	5/4222282	5	4222282	Intercross	C/T	2.13 × 10^−4^	Intergenic
4	5/4226091	5	4226091	Intercross	C/T	9.90 × 10^−5^	Intergenic
5	5/4804826	5	4804826	Intercross	G/C	2.62 × 10^−4^	Exon, POPTR_0005s07150.1 CBS/octicosapeptide/Phox/ Bemp1 (PB1) domain‐containing protein AT3G52950.1
6	7/13898377	7	13898377	Intercross	T/C	3.05 × 10^−4^	Exon, POPTR_0007s13900.1 3‐oxo‐5‐alpha‐steroid 4‐dehydrogenase family protein AT2G16530.1
7	8/10163499	8	10163499	Intercross	G/T	2.69 × 10^−4^	Intergenic, POPTR_0008s15190.1 Ribosomal protein S24e family protein AT5G28060.1
8	9/8371476	9	8371476	Intercross	A/T	2.41 × 10^−4^	Intergenic, POPTR_0009s09470.1 Protein phosphatase 2C family protein AT4G33920.1
9	14/763510	14	763510	Intercross	G/T	3.14 × 10^−4^	Intergenic
10	14/17660801	14	17660801	Intercross	A/G	2.25 × 10^−4^	Intergenic
11	16/12803482	16	12803482	Intercross	C/A	3.14 × 10^−4^	Intergenic, POPTR_0016s13640.1 AT2G38450.1

**Figure 4:**
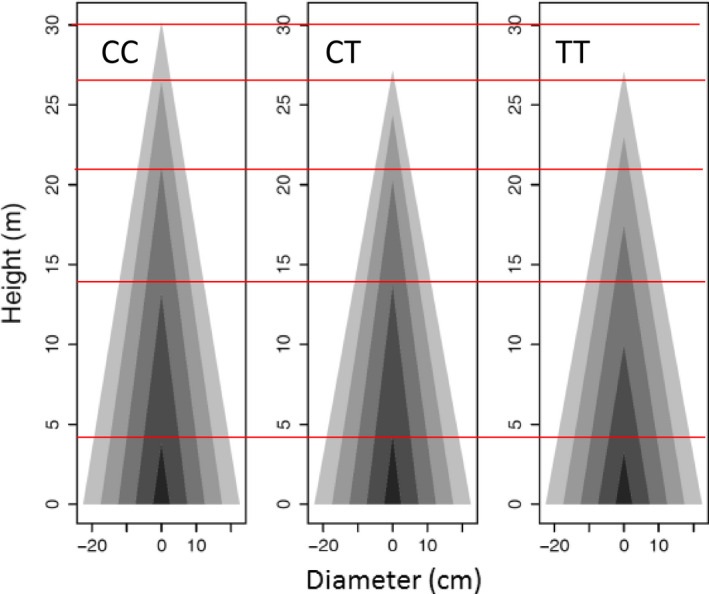
Developmental allometry of how stem height scales with stem diameter, modulated by *pi* QTL 5/4226091 with three different genotypes CC, CT and TT. Red lines show the differences of stem height growth among three genotypes at five representative ages.

Based on estimated genotype‐specific mathematical parameters *a*_*jH*←*D*_ and *b*_*jH*←*D*_ from equation (1a) (Table S1), we can draw the profile of stem height and diameter growth. Genotype CC at *pi*QTL 5/4226091 is taller than genotypes CT and TT when they achieve the same diameter at a late stage of growth (Figure 4). However, in early stages, genotype CC and CT are similar in height–diameter allometry, taller than genotype TT of the same diameter. These suggest that this *pi*QTL exerts a time‐varying effect on stem form during ontogeny. Similar phenomena can be observed for the other *pi*QTLs.

#### MiQTLs: How diameter growth scales with height growth

**Figure 5:**
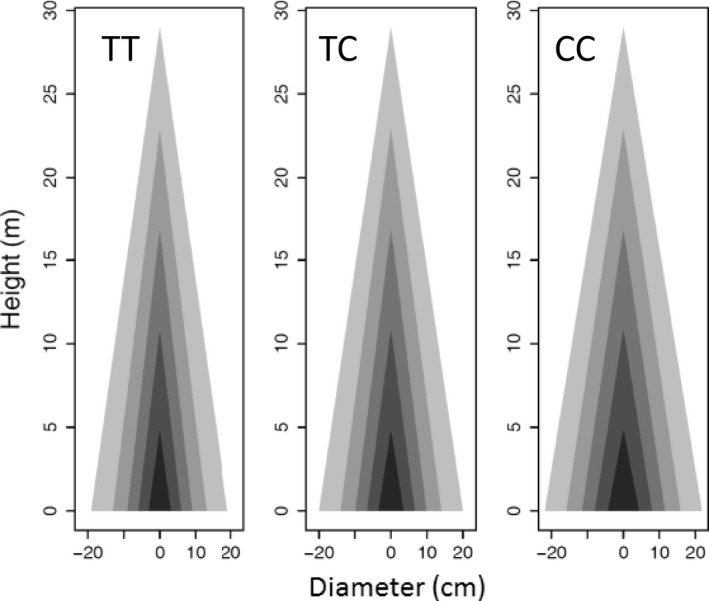
Developmental allometry of how stem diameter scales with stem height, modulated by *mi* QTL 5/4220981 with three different genotypes TT, TC and CC. Genotype CC is much thicker than the other two (TT, TC) per the same amount of stem height growth.

Functional mapping based on equation (1b) identified 11 significant *mi*QTLs that determine the allomteric scaling of stem diameter with stem height (Table 1; Figure 2b), of which one is distributed on chromosome 7, 8, 9 and 16, two distributed on chromosome 14 and five distributed on chromosome 5. It can be seen that the detected *mi*QTLs can well fit the raw data of stem diameter scaling with height (Figure S2). Genetic effects on radial growth, including additive and dominant effects, at all *mi*QTLs identified increase consistently with stem growth (Figure 3b), but there is substantial difference in the trajectory of additive and dominant effects at the same *Mi*QTL. For example, the additive curve of *mi*QTL 5/4220981 on chromosome 5 is convex with height growth, whereas its dominant curve is concave. Estimated parameters (*a*_*D*←*H*_ and *b*_*D*←*H*_) from equation (1b) (Table S2) were used to draw the profiles of tree stem for individual genotypes. With the same stem height, genotype CC at *mi*QTL 5/4220981 is stouter than genotype CT, which is stouter than genotype TT (Figure 5). This shows that this *mi*QTL determines the capacity of a tree to maintain its competitive advantage in a crowd stand. Differences in stem profiles between different genotypes can also be seen at the other *mi*QTLs.

It is interesting to note that there are four *Pi*QTLs each that also act as a *mi*QTL; that is, these QTLs simultaneously govern the allometric scaling of stem height with stem diameter and the allometric scaling of stem diameter with stem height. These shared QTLs are located on chromosome 8 (8/10163499), chromosome 9 (9/8371476) and chromosome 14 (14/763510 and 14/17660801). As these two aspects of allometry present different biological processes, the QTLs detected for both types are thought to be pleiotropic. However, their effects on the two aspects of H‐D allometry were found to display different significance levels, suggesting the existence of QTL–development interactions. It is interesting to see that *mi*QTL 5/4226091 is very close to *mi*QTL 5/4220981 in a similar region of chromosome 5, suggesting that their linkage may be a cause of a tree's pioneering and maintaining capacity for growth resource.

#### Functional annotation of QTLs

A critical issue about results from genetic mapping is how to interpret the biological function of significant QTLs detected. Among the *pi*QTLs detected to affect the developmental allometry of height with diameter by functional mapping, more than half (9/16) are located near candidate genes for cell metabolism, growth and differentiation (Table 1). For example, *pi*QTL 8/10163499 is in the proximity of genes that encode ribosomal protein S24e family, and *pi*QTL 14/791796 is located in the gene family that encodes P‐loop containing nucleoside triphosphate hydrolases superfamily protein. Six of the 11 *mi*QTLs are related to candidate of known function (Table 1). For example, *Mi*QTL 5/4804826 is near the gene that encodes CBS/octicosapeptide/Phox/Bemp1 (PB1) domain‐containing protein. The biological function of those QTLs whose annotations are not available remains to be validated.

## Discussion

Tree height for a given diameter may vary strikingly among species (King, 1996) and across biogeographical regions (Feldpausch *et al*., 2011; Hulshof *et al*., 2015). Such variation could hold important implications for carbon storage potential of forests, but their underlying genetic control mechanisms has been long a puzzle. Currently, emerging genomewide association studies are equipped with a capacity to unravel a complete resolution of the genetic architecture of complex traits by identifying individual genes involved in phenotypic variation, their number, locations, actions and interactions, and pleiotropy (Wang *et al*., 2005). Given that any trait undergoes a dynamic process, the integration of developmental processes that give rise to a final phenotype into association studies has become essential, in order to better chart a genotype–phenotype map (Salazar‐Ciudad and Marin‐Riera, 2013). The basic principle and procedure for such integration have been recorded in functional mapping (Wu and Lin, 2006) or functional genomewide association studies (*f*GWAS) (Das *et al*., 2011). By utilizing mathematical aspects of trait formation and development, these approaches have been shown to be both statistically powerful and biologically relevant (Li and Sillanpää, 2016).

This study presents the application of functional mapping to unveil the genetic architecture of stem height–diameter allometry by integrating biologically meaningful equations of this phenomenon (Henry and Aarssen, 1999; Huang *et al*., 1992; Hulshof *et al*., 2015). This new application can not only characterize how individual genes affect dynamic variation in stem apical and radial growth, but also explore how these two growth processes are related by pleiotropic genes or different genes in a strong linkage. In nature, stem height–diameter relationship is a predictor of species' geographical distribution and ecosystem functioning (Feldpausch *et al*., 2011; Hulshof *et al*., 2015). Better height growth for a given diameter enables trees to acquire an advantage of competing for sunlight (Moles *et al*., 2009). Thus, any genes that modulate the change in stem height as a function of diameter are likely to participate in the pioneering process of space occupation. By analysing a GWAS data of poplar hybrids grown in a field trial, functional mapping shows it unique power to identify and map these so‐called pioneering QTLs (*pi*QTLs). We have found 16 such *pi*QTLs that mediate the allometric change in stem height with stem diameter, some of which have been validated in terms of their biological function.

After trees obtain a spatial advantage, how to maintain this advantage through strengthening mechanical support (McMahon, 1973; Niklas, 1993) and water transport efficiency (Bullock, 2000) will quickly become their next task in order to maximize the fitness of trees. More rapid stem radial growth for a given stem height can facilitate the maintenance of competing advantage. Therefore, any genes that are associated with the change in stem diameter growth with stem height may play an important role in modulating the investment of energy in structural support. A total of 11 such so‐called maintaining QTLs (*mi*QTLs) have been identified by *f*GWAS from the poplar GWAS experiment. Unlike *pi*QTLs that operate mostly before the emergence of spatial competition, these *mi*QTLs may be activated after competition sets in the stand. However, *pi*QTLs and *mi*QTLs are not exclusive from each other because many of them pleiotropically affect both the allometric scaling of stem height with stem diameter and the allometric scaling of stem diameter with stem height, although QTL–development interactions are also identified.

In general, the expected sizes of genetic effects on a complex trait are small and, also, the expected number of false‐positive findings can be large so that large sample sizes and replication samples are needed (Wang *et al*., 2005). However, unlike other small‐sized and well‐funded organisms, it is practically challenging to obtain large sample sizes for forest trees. Forest trees have many desirable properties which can compensate for a small sample size. Through intra‐ or interspecific cross, they can generate a full‐sib family with homogeneous genetic background, requiring no extra samples needed to remove the influence of population structure and other covariates that leads to spurious associations as seen in human genomewide association studies (Manolio, 2010). The study material of forest trees can be grown in a uniform environment and allow destructive sampling for precise phenotyping, which increases the heritability of a complex trait and, therefore, the power of gene identification, by reducing environmental noises and measurement errors. These advantages, plus repeated measurement of the same phenotype at a series of time points, can augment the amount of informativeness involved in trait variation for forest tree association studies.

This article describes and demonstrates a study of using functional mapping to map growth allometry. The idea behind the study deserves a widespread application to more comprehensive experiments. For example, our current study was established in a single environment, but this is obviously inadequate to study the genetic control of how height–diameter allometry responds to changing environment. However, possible vegetative propagation of trees allows the study population to be replicated in time and space, enabling the dissection of genetic and environmental contributions to developmental variation (Wu, 1996). In particular, since height–diameter allometry is sensitive to density, field studies laid with multiple sets of spacing using clonal replicates allow the identification of how *pi*QTLs or *mi*QTLs alter their expression in response to the change in environmental conditions like sunlight and resources.

Association studies based on a simple regression approach may be limited for statistical inference about the genetic basis of quantitative traits. By simultaneously analysing all possible SNPs, this issue can be resolved from a variable selection model, such as LASSO, allowing a subset of predictors to be identified from genetic data in which the number of variables is far larger than the number of samples (Li *et al*., 2011; Mutshinda and Sillanpaa, 2010). More recently, Li *et al*. (2015) has incorporated variable selection into *f*GWAS, allowing geneticists to chart a global picture of genetic control over developmental processes of any complex traits.

## Experimental procedures

### Mapping design

We generated a mapping population composed of full‐sibs derived from interspecific cross between female clone I‐69 of cottonwood (*Populus deltoides*) introduced from the United State and male clone I‐45 of *P. ×euramerican*, a hybrid between *P. deltoides* and *P. nigra* originally from Europe. In 1987, this family containing 450 hybrid trees was planted with ramets in a uniform site in terms of soil physical properties, moisture and nutrient contents at Zhangji Forest Farm, Xuzhou, Jiangsu. After growing in the field for 24 years, part of the family (64 hybrids and two parents, I‐69 and I‐45) was harvested for stem analysis, from which annual growth for stem height and diameter was measured.

The harvested trees were genotyped at SNPs for genomewide mapping. Genotyping was performed on the Applied Biosystems^™^ QuantStudio^™^ 12K Flex Real‐Time PCR System. SNP genotypes were obtained after stringent quality‐control filters. To the end, a total of 156 362 segregating SNPs were obtained. Of these, 94 591 and 61 771 belongs to the testcross and intercross markers, respectively. In a full‐sib family derived from two heterozygous tree parents, there are two possible marker types that are segregating with different patterns: (i) testcross markers at which one parent is heterozygous but the other is homozygous, and (ii) intercross markers at which both parents are heterozygous (Lu *et al*., 2004; Malieparrd *et al*., 1997; Tong *et al*., 2011; Wu *et al*., 2002).

We assume that all progeny are planted in a randomized design in a field trial, measured longitudinally for both stem height and diameter growth at a multitude of ages during ontogeny. From this experiment, we obtain both marker and dynamic phenotypic data (with *T* time points) whose structure is schematically shown in Table 2, where we will test whether any of these markers (denoted as 1, …, *p*) is significantly associated with stem growth phenotype. Traditional approaches test the significance of association between marker genotypes and phenotypes measured at a static time. Functional mapping breaks through the limitation of traditional approaches by associating marker genotypes with longitudinal phenotypes across a series of time points. This treatment allows the characterization of how a QTL governs the dynamic change in phenotypic traits. Here, we show that functional mapping can be extended to map dynamic trait–trait relationships during ontogeny, which can shed light on the pleiotropic control mechanisms of trait correlations.

**Table 2:** Marker and phenotypic data structure of a full‐sib family derived from two parents P_1_ and P_2_. Height and diameter covary over age in equation (1a) or (1b)

	No.						Longitudinal phenotype					
		Marker					Height			Diameter		
		1	2	3	…	*p*	1	…	*T*	1	…	*T*
Parent	P_1_	*AA*	*Aa*	*Aa*	*…*	*Aa*	*y*_P1_(1)	…	*y*_P1_(*T*)	*z*_P1_(1)	…	*z*_P1_(*T*)
	P_2_	*Aa*	*AA*	*Aa*	*…*	*Aa*	*y*_P2_(1)	…	*y*_P2_(*T*)	*z*_P2_(1)	…	*z*_P2_(*T*)
Progeny	1	*AA*	*Aa*	*aa*	*…*	*AA*	*y*_1_(1)	…	*y*_1_(*T*)	*z*_1_(1)	…	*z*_1_(*T*)
	2	*AA*	*AA*	*Aa*	*…*	*Aa*	*y*_2_(1)	…	*y*_2_(*T*)	*z*_2_(1)	…	*z*_2_(*T*)
	3	*Aa*	*Aa*	*Aa*	*…*	*Aa*	*y*_3_(1)	…	*y*_3_(*T*)	*z*_3_(1)	…	*z*_3_(*T*)
	4	*AA*	*Aa*	*aa*	*…*	*Aa*	*y*_4_(1)	…	*y*_4_(*T*)	*z*_4_(1)	…	*z*_4_(*T*)
	5	*Aa*	*Aa*	*AA*	*…*	*aa*	*y*_5_(1)	…	*y*_5_(*T*)	*z*_5_(1)	…	*z*_5_(*T*)
	6	*Aa*	*AA*	*AA*	*…*	*Aa*	*y*_6_(1)	…	*y*_6_(*T*)	*z*_6_(1)	…	*z*_6_(*T*)
	7	*AA*	*AA*	*Aa*	*…*	*aa*	*y*_7_(1)	…	*y*_7_(*T*)	*z*_7_(1)	…	*z*_7_(*T*)
	8	*Aa*	*Aa*	*Aa*	*…*	*Aa*	*y*_8_(1)	…	*y*_8_(*T*)	*z*_8_(1)	…	*z*_8_(*T*)
	⋮											
	*n*	*Aa*	*AA*	*Aa*	*…*	*AA*	*y*_*n*_(1)	…	*y*_*n*_(*T*)	*z*_*n*_(1)	…	*z*_*n*_(*T*)

### Functional mapping for H‐D allometry

Tremendous efforts have been made to quantify the allometric relationship of stem height and diameter by deriving robust mathematical equations (Henry and Aarssen, 1999; Huang *et al*., 1992; Hulshof *et al*., 2015). One example of describing how stem height and diameter covary for popular species is expressed as

(1a) $$H\left(t\right)=H_{0}+e^{a_{H\leftarrow D}+\frac{1}{b_{H\leftarrow D}+D\left(t\right)}}$$

(1b) $$D\left(t\right)=\frac{1-\left[\ln\left(H\left(t\right)-H_{0}\right)-a_{D\leftarrow H}\right]b_{D\leftarrow H}}{\ln\left(H\left(t\right)-H_{0}\right)-a_{D\leftarrow H}}$$

where *H*(*t*) and *D*(*t*) are the stem height and diameter of a given tree at time *t*; H_0_ is the stem height in the establishment year; and *a*_*H*←*D*_ and *b*_*H*←*D*_ are two different power coefficients of stem height scaling with stem diameter in forest trees, whereas *a*_*D*←*H*_ and *b*_*D*←*H*_ are those of stem diameter scaling with height. Although equations (1a) and (1b) are mathematically interchangeable in describing the H‐D relationship, they differ in the direction of allometric scaling. Equation (1a) describes how stem height increases in response to the increase in stem diameter through parameters *a*_*H*←*D*_ and *b*_*H*←*D*_, whereas equation (1b) specifies the impact of stem height on radial growth during ontogeny through parameters *a*_*D*←*H*_ and *b*_*D*←*H*_. As will be shown below, (*a*_*H*←*D*_, *b*_*H*←*D*_) and (*a*_*D*←*H*_, *b*_*D*←*H*_) are two different sets of parameters, if the genotypic value of a QTL as a response at the left side of equation (1a) or (1b) is predicted by the phenotypic value as a dependent variable at the right side.

The integration of functional mapping into a QTL mapping study of H‐D allometry can be made through a multiplicative likelihood of different marker genotypes (Das *et al*., 2011). As all progeny are from a single full‐sib family, they share the same mutual relationship based on quantitative genetic theory. Let y_*i*_ = (*y*_*i*_(1), …, *y*_*i*_(*T*) and z_*i*_ =  (*z*_*i*_(1), …, *z*_*i*_(*T*)) denote stem height and diameter growth over a series of T time points, respectively. Their likelihoods are formulated as

(2a) $$L\left(y\right)=\prod_{j=1}^{J}\prod_{i=1}^{n_{j}}f_{j}^{H}\left(y_{i};\mu_{j|i}^{H},\Sigma_{i}^{H}\right),$$

(2b) $$L\left(z\right)=\prod_{j=1}^{J}\prod_{i=1}^{n_{j}}f_{j}^{D}\left(z_{i};\mu_{j|i}^{D},\Sigma_{i}^{D}\right),$$

where *j* stands for the *j*th genotype of a SNP (*j *=* *1, …, *J*), *n*_*j*_ is the observation of the *j*th genotype; and $f_{j}^{H}\left(y_{i};\mu_{j|i}^{H},\Sigma_{i}^{H}\right)$ and $f_{j}^{D}\left(z_{i};\mu_{j|i}^{D},\Sigma_{i}^{D}\right)$ are multivariate normal distributions of stem height and diameter, respectively, with expected mean vector of genotype *j* for progeny *i* over *T* time points, expressed as

(3a) $$\mu_{j|i}^{H}=\left(\mu_{j|i}^{H}\left(1\right),\ldots ,\mu_{j|i}^{H}\left(T\right)\right)$$

(3b) $$\mu_{j|i}^{D}=\left(\mu_{j|i}^{D}\left(1\right),\ldots ,\mu_{j|i}^{D}\left(T\right)\right)$$

and (*T *× *T*) longitudinal matrix composed of time‐dependent variances and covariances expressed as

(4a) $$\Sigma_{i}^{H}=\left(\begin{array}{ccc} \sigma_{H}^{2}\left(1\right) & \cdots & \sigma_{H}\left(1,T\right)\\ \vdots & \ddots & \vdots \\ \sigma_{H}\left(T,1\right) & \cdots & \sigma_{H}^{2}\left(T\right) \end{array}\right)$$

(4b) $$\Sigma_{i}^{D}=\left(\begin{array}{ccc} \sigma_{D}^{2}\left(1\right) & \cdots & \sigma_{D}\left(1,T\right)\\ \vdots & \ddots & \vdots \\ \sigma_{D}\left(T,1\right) & \cdots & \sigma_{D}^{2}\left(T\right) \end{array}\right)$$

Functional mapping models the mean vector (2a) and (2b) by a biologically meaningful mathematical equation, such as allometry growth equation (1a) or (1b), which is expressed as

(5a) $$\begin{array}{cc} \mu_{j|i}^{H} & =\left(\mu_{j|i}^{H}\left(1\right),\ldots ,\mu_{j|i}^{H}\left(T\right)\right)\\ & =\left(H_{i0}+e^{a_{jH\leftarrow D}+\frac{1}{b_{jH\leftarrow D}+z_{i}\left(1\right)}},\ldots ,H_{i0}+e^{a_{jH\leftarrow D}+\frac{1}{b_{jH\leftarrow D}+z_{i}\left(T\right)}}\right) \end{array}$$

(5b) $$\mu_{j|i}^{D}=\left(\mu_{j|i}^{D}\left(1\right),\ldots ,\mu_{j|i}^{D}\left(T\right)\right)=\left(\frac{1-\left[\ln\left(y_{i}\left(1\right)-H_{i0}\right)-a_{jD\leftarrow H}\right]b_{jD\leftarrow H}}{\ln\left(y_{i}\left(1\right)-H_{i0}\right)-a_{jD\leftarrow H}},\ldots ,\frac{1-\left[\ln\left(y_{i}\left(T\right)-H_{i0}\right)-a_{jD\leftarrow H}\right]b_{jD\leftarrow H}}{\ln\left(y_{i}\left(T\right)-H_{i0}\right)-a_{jD\leftarrow H}}\right)$$

where power parameters (*a*_*jH*←*D*_, *b*_*jH*←*D*_) and (*a*_*jD*←*H*_, *b*_*jD*←*H*_) are defined for genotype *j*. Equations (5a) or (5b) indicates that the genotypic value of one growth trait at a time is modelled by the phenotypic value of its allometrically related trait. This can be more generally expressed as

(6a) $$\mu_{j|i}^{H}\left(t\right)=h\left(a_{jH\leftarrow D},b_{jH\leftarrow D};\mu_{j|i}^{D}\left(t\right)+e_{i}^{D}\left(t\right)\right)$$

(6b) $$\mu_{j|i}^{D}\left(t\right)=d\left(a_{jD\leftarrow H},b_{jD\leftarrow H};\mu_{j|i}^{H}\left(t\right)+e_{i}^{H}\left(t\right)\right)$$

where $\mu_{j|i}^{H}\left(t\right)$ and $\mu_{j|i}^{D}\left(t\right)$ are the genotypic value of stem height and diameter at time *t*, respectively, which is a function of the phenotypic value of its allomteric trait in form *h* or *d*, respectively, and $e_{i}^{H}\left(t\right)$ and $e_{i}^{D}\left(t\right)$ are the residual errors of these two traits at time *t*, with a longitudinal covariance matrix (4a) or (4b). Because $e_{i}^{H}\left(t\right)$ and $e_{i}^{D}\left(t\right)$ have different structures, parameters (*a*_*jH*←*D*_, *b*_*jH*←*D*_) and (*a*_*jD*←*H*_, *b*_*jD*←*H*_) estimated from functions *h* and *d* should be different even though these two functions are interchangeable.

Functional mapping also models the covariance matrices (4a) and (4b) by a parsimonious and flexible approach, such as an autoregressive (Ma *et al*., 2004), antedependence (Zhao *et al*., 2005), autoregressive moving average (Li *et al*., 2010) or nonparametric and semiparametric approaches (Das *et al*., 2011). The first‐order autoregressive (AR(1)) approach is computationally efficient, but needs the stationarity assumption. Compared with this approach, the others are more flexible, but may be computationally more expensive. By analysing the raw data, it was observed that diameter‐dependent variation in height (Figure 1a) and height‐dependent variation in diameter (Figure 1b) are quite stationary over the dependent variable. Thus, the computationally efficient AR(1) approach was used to model the covariance structure of matrices (4a) and (4b).

Functional mapping has been implemented with several statistical algorithms for obtaining the maximum likelihood estimates (MLEs) of genotype‐specific growth parameters and the parameters that model the covariance structure. These algorithms include the EM algorithm and some optimization techniques, like the simplex algorithm (Ma *et al*., 2002; Zhao *et al*., 2005).

### Testing pioneering QTLs or maintaining QTLs

To test whether a particular SNP is associated with height–diameter growth allometry, we just need to test whether a set of power parameters (*a*_*jH*←*D*_, *b*_*jH*←*D*_) or (*a*_*jD*←*H*_, *b*_*jD*←*H*_) differs jointly among genotypes. This can be done, respectively, by formulating the following two sets of hypotheses:

(7a) $$\begin{array}{cc} & H_{0}:\left(a_{jH\leftarrow D},b_{jH\leftarrow D}\right)\equiv \left(a_{H\leftarrow D},b_{H\leftarrow D}\right),\;\text{for all}\;j=1,\ldots ,J\\ & H_{1}:\;\text{At least one of the equalities in the}\;\;H_{0}\;\text{does not hold} \end{array}$$

(7b) $$\begin{array}{cc} & H_{0}:\left(a_{jD\leftarrow H},b_{jD\leftarrow H}\right)\equiv \left(a_{D\leftarrow H},b_{D\leftarrow H}\right),\;\text{for all}\;j=1,\ldots ,J\\ & H_{1}:\;\text{At least one of the equalities in the}\;\;H_{0}\;\text{does not hold} \end{array}$$

After the MLEs of the unknown parameters under the H_0_ and H_1_ for each test are obtained, the log‐likelihood ratio (LR) is calculated. By comparing it with the critical threshold of a chi‐square distribution, the *P*‐value reflecting the significance of the SNP considered is then obtained. The approach for adjusting for multiple comparisons, such as Bonferroni, to take into account all SNPs was used to obtain a genomewide threshold. The SNPs whose significance level is beyond the adjusted criterion are regarded as QTLs. The significance tests based on hypotheses (7a) and (7b) produce ecologically different interpretations. Hypothesis (7a) intends to find a QTL that modulates how height growth increases per diameter growth, a process critically determining a tree's capacity to pioneer growth space. Such a QTL is called the ‘pioneering’ QTL or piQTLs. On the other hand, hypothesis (7b) can detect the so‐called maintaining QTL or miQTLs that regulates the increase of stem girth per height growth, which is related to the capacity of a tree to maintain growth space.

## Supporting information


**Figure S1** Genotypic curves (in different colors) at *pi*QTL.that modulate the allometry of tree height with stem diameter.
**Figure S2** Genotypic curves (in different colors) at *mi*QTL.that modulate the allometry of tree height with stem diameter.

**Table S1** MLEs of parameters for *pi*QTLs that model the allometry of tree height with stem diameter by equations (1a).
**Table S2** MLEs of parameters for *mi*QTLs that model the allometry of tree height with stem diameter by equations (1b).
