# Supplementary material for: Computational identification of genes modulating stem height–diameter allometry
Source: Plant Biotechnol J. 2016 Jun 15;14(12):2254–64. doi: 10.1111/pbi.12579 (PMC5103235; doi:10.1111/pbi.12579)
Supplement: Supplementary file 1 — Figure S1 Genotypic curves (in different colors) at piQTL.that modulate the allometry of tree height with stem diameter. Figure S2 Genotypic curves (in different colors) at miQTL.that modulate the allometry of tree height with stem diameter. [file PBI-14-2254-s002.doc]

**Figure S1** Genotypic curves (in different colors) at *p*QTL.that modulate the allometry of tree height with stem diameter in a full-sib family of poplars. The curves in grey are those of 62 poplar hybrids.

**Figure S2** Genotypic curves (in different colors) at *m*QTL.that modulate the allometry of stem diameter with tree height in a full-sib family of poplars. The curves in grey are those of 62 poplar hybrids.
