# Supplementary material for: Computational identification of genes modulating stem height–diameter allometry
Source: Plant Biotechnol J. 2016 Jun 15;14(12):2254–64. doi: 10.1111/pbi.12579 (PMC5103235; doi:10.1111/pbi.12579)
Supplement: Supplementary file 2 — Table S1 MLEs of parameters for piQTLs that model the allometry of tree height with stem diameter by equations (1a). Table S2 MLEs of parameters for miQTLs that model the allometry of tree height with stem diameter by equations (1b). [file PBI-14-2254-s001.docx]

Table S1 MLEs of parameters for piQTLs that model the allometry of tree height with stem diameter by equations (1a). The standard errors of the MLEs are given parentheses.

| $H(t)=H_{0}+e^{a_{H\leftarrow D}+\frac{1}{b_{H\leftarrow D}+D(t)}}$ | | | | | | | | | | | |
| --- | --- | --- | --- | --- | --- | --- | --- | --- | --- | --- | --- |
| SNP |  | Chr. | Position | QQ | | Qq | | qq | | AR(1) | |
|  |  |  |  | a_1H←D_ | b_1H←D_ | a_2H←D_ | b_2H←D_ | a_3H←D_ | b_3H←D_ | φ | ν^2^ |
| 1 |  | 5 | 4226091 | 3.82 | -23.89 | 3.62 | -19.85 | 3.84 | -30.29 | 0.84 | 0.69 |
|  |  |  |  | (0.097) | (2.626) | (0.063) | (1.777) | (0.089) | (2.656) | (0.009) | (0.040) |
| 2 |  | 5 | 24780848 | 3.83 | -29.74 | 3.64 | -19.79 | 3.76 | -25.71 | 0.84 | 0.68 |
|  |  |  |  | (0.090) | (2.662) | (0.052) | (1.511) | (0.092) | (2.608) | (0.008) | (0.045) |
| 3 |  | 6 | 17682099 | 3.69 | -22.85 | 3.67 | -20.65 | 3.92 | -32.50 | 0.84 | 0.69 |
|  |  |  |  | (0.094) | (2.519) | (0.069) | (1.934) | (0.119) | (3.739) | (0.007) | (0.046) |
| 4 |  | 8 | 10141590 | 3.81 | -27.92 | 3.52 | -16.63 | 3.76 | -25.78 | 0.84 | 0.70 |
|  |  |  |  | (0.107) | (3.443) | (0.068) | (1.703) | (0.094) | (2.911) | (0.008) | (0.047) |
| 5 |  | 8 | 10163499 | 3.64 | -19.30 | 3.80 | -27.51 | 3.56 | -17.51 | 0.84 | 0.68 |
|  |  |  |  | (0.091) | (2.207) | (0.065) | (2.017) | (0.073) | (1.903) | (0.009) | (0.040) |
| 6 |  | 8 | 10214577 | 3.80 | -27.61 | 3.56 | -17.64 | 3.73 | -25.04 | 0.84 | 0.68 |
|  |  |  |  | (0.110) | (3.344) | (0.061) | (1.537) | (0.096) | (2.954) | (0.008) | (0.041) |
| 7 |  | 8 | 10214969 | 3.77 | -26.83 | 3.58 | -18.06 | 3.85 | -28.55 | 0.84 | 0.69 |
|  |  |  |  | (0.102) | (3.214) | (0.064) | (1.463) | (0.097) | (3.115) | (0.007) | (0.041) |
| 8 |  | 8 | 10386508 | 3.68 | -24.29 | 3.58 | -17.93 | 3.74 | -26.33 | 0.84 | 0.69 |
|  |  |  |  | (0.088) | (2.910) | (0.069) | (1.810) | (0.107) | (3.317) | (0.006) | (0.045) |
| 9 |  | 9 | 8371476 | 3.82 | -29.07 | 3.57 | -18.63 | 3.60 | -19.23 | 0.84 | 0.69 |
|  |  |  |  | (0.091) | (2.839) | (0.065) | (1.826) | (0.116) | (2.824) | (0.008) | (0.043) |
| 10 |  | 11 | 645420 | 3.61 | -20.68 | 3.69 | -21.32 | 3.86 | -30.81 | 0.84 | 0.65 |
|  |  |  |  | (0.096) | (2.956) | (0.058) | (1.579) | (0.108) | (3.440) | (0.007) | (0.038) |
| 11 |  | 14 | 139257 | 3.68 | -19.18 | 3.64 | -23.23 | 3.79 | -24.84 | 0.84 | 0.69 |
|  |  |  |  | (0.069) | (1.659) | (0.069) | (2.225) | (0.083) | (2.412) | (0.008) | (0.041) |
| 12 |  | 14 | 763510 | 3.63 | -22.46 | 3.62 | -19.34 | 3.81 | -28.48 | 0.84 | 0.69 |
|  |  |  |  | (0.107) | (2.830) | (0.056) | (1.570) | (0.097) | (2.960) | (0.008) | (0.041) |
| 13 |  | 14 | 791796 | 3.81 | -25.14 | 3.66 | -23.90 | 3.64 | -18.56 | 0.84 | 0.69 |
|  |  |  |  | (0.080) | (2.308) | (0.057) | (1.838) | (0.050) | (1.353) | (0.008) | (0.042) |
| 14 |  | 14 | 807942 | 3.62 | -23.06 | 3.60 | -19.50 | 3.77 | -27.45 | 0.84 | 0.70 |
|  |  |  |  | (0.124) | (3.678) | (0.063) | (1.566) | (0.096) | (2.848) | (0.008) | (0.040) |
| 15 |  | 14 | 17660801 | 3.71 | -24.36 | 3.63 | -19.64 | 3.91 | -32.16 | 0.84 | 0.69 |
|  |  |  |  | (0.081) | (2.478) | (0.070) | (1.989) | (0.132) | (4.416) | (0.008) | (0.048) |
| 16 |  | 16 | 11391387 | 3.68 | -25.39 | 3.63 | -20.12 | 3.97 | -31.82 | 0.84 | 0.69 |
|  |  |  |  | (0.127) | (3.791) | (0.050) | (1.333) | (0.111) | (3.372) | (0.007) | (0.042) |

Table S2 MLEs of parameters for miQTLs that model the allometry of tree height with stem diameter by equations (1a). The standard errors of the MLEs are given parentheses.

| $D(t)=\frac{1-[\ln(H(t)-H_{0})-a_{D\leftarrow H}]b_{D\leftarrow H}}{\ln(H(t)-H_{0})-a_{D\leftarrow H}}$ | | | | | | | | | | | |
| --- | --- | --- | --- | --- | --- | --- | --- | --- | --- | --- | --- |
|  | SNP | Chr. | Position | QQ | | Qq | | qq | |  | |
|  |  |  |  | a_1D←H_ | b_1D←H_ | a_2D←H_ | b_2D←H_ | a_3D←H_ | b_3D←H_ | φ | ν^2^ |
| 1 |  | 5 | 4220981 | 4.01 | -32.74 | 3.87 | -26.09 | 3.91 | -24.29 | 0.83 | 1.34 |
|  |  |  |  | (0.059) | (2.199) | (0.052) | (1.565) | (0.069) | (1.955) | (0.009) | (0.071) |
| 2 |  | 5 | 4221532 | 4.03 | -33.79 | 3.86 | -25.47 | 3.90 | -24.48 | 0.83 | 1.33 |
|  |  |  |  | (0.065) | (2.386) | (0.062) | (1.673) | (0.086) | (2.427) | (0.011) | (0.090) |
| 3 |  | 5 | 4222282 | 3.91 | -25.17 | 3.88 | -25.88 | 4.04 | -34.56 | 0.83 | 1.35 |
|  |  |  |  | (0.073) | (2.165) | (0.046) | (1.533) | (0.081) | (3.035) | (0.009) | (0.086) |
| 4 |  | 5 | 4226091 | 3.88 | -23.78 | 3.83 | -24.11 | 3.97 | -32.36 | 0.83 | 1.34 |
|  |  |  |  | (0.101) | (3.009) | (0.041) | (1.363) | (0.066) | (2.491) | (0.010) | (0.086) |
| 5 |  | 5 | 4804826 | 3.86 | -22.86 | 3.89 | -27.74 | 4.02 | -32.67 | 0.83 | 1.29 |
|  |  |  |  | (0.062) | (1.683) | (0.050) | (1.576) | (0.065) | (2.540) | (0.010) | (0.094) |
| 6 |  | 7 | 13898377 | 3.79 | -23.65 | 3.96 | -27.55 | 3.85 | -29.83 | 0.83 | 1.32 |
|  |  |  |  | (0.070) | (2.350) | (0.046) | (1.320) | (0.068) | (2.615) | (0.009) | (0.069) |
| 7 |  | 8 | 10163499 | 3.86 | -23.62 | 3.99 | -31.44 | 3.91 | -25.41 | 0.83 | 1.33 |
|  |  |  |  | (0.082) | (2.305) | (0.042) | (1.605) | (0.072) | (1.994) | (0.009) | (0.070) |
| 8 |  | 9 | 8371476 | 3.99 | -32.67 | 3.93 | -26.57 | 3.82 | -24.14 | 0.83 | 1.35 |
|  |  |  |  | (0.055) | (2.062) | (0.051) | (1.754) | (0.064) | (1.716) | (0.009) | (0.073) |
| 9 |  | 14 | 763510 | 4.08 | -33.29 | 3.87 | -24.75 | 3.91 | -30.49 | 0.83 | 1.35 |
|  |  |  |  | (0.111) | (3.184) | (0.041) | (1.347) | (0.056) | (1.934) | (0.008) | (0.083) |
| 10 |  | 14 | 17660801 | 3.84 | -26.50 | 3.90 | -25.11 | 4.02 | -34.10 | 0.83 | 1.35 |
|  |  |  |  | (0.068) | (2.498) | (0.045) | (1.452) | (0.093) | (3.295) | (0.009) | (0.086) |
| 11 |  | 16 | 12803482 | 4.00 | -33.25 | 3.85 | -24.36 | 4.06 | -33.11 | 0.83 | 1.33 |
|  |  |  |  | (0.088) | (3.168) | (0.043) | (1.429) | (0.070) | (2.422) | (0.010) | (0.083) |
